# A Digital Tool for the Self-Assessment of Homes to Increase Age-Friendliness: Validity Study

**DOI:** 10.2196/49500

**Published:** 2023-10-26

**Authors:** Roslyn Aclan, Stacey George, Kate Laver

**Affiliations:** 1 College of Medicine and Public Health Flinders University of South Australia Adelaide Australia; 2 Caring Futures Institute, College of Nursing and Health Sciences Flinders University of South Australia Adelaide Australia

**Keywords:** age-friendliness, aging, home environment, self-assessment, digital, tool

## Abstract

**Background:**

Age-friendly environments in homes and communities play an important role in optimizing the health and well-being of society. Older people have strong preferences for remaining at home as they age. Home environment assessment tools that enable older people to assess their homes and prepare for aging in place may be beneficial.

**Objective:**

This study aims to establish the validity of a digital self-assessment tool by assessing it against the current gold standard, an occupational therapy home assessment.

**Methods:**

A cohort of adults aged ≥60 years living in metropolitan Adelaide, South Australia, Australia, assessed their homes using a digital self-assessment tool with 89 questions simultaneously with an occupational therapist. Adults who were living within their homes and did not have significant levels of disabilities were recruited. Cohen κ and Gwet AC_1_ were used to assess validity.

**Results:**

A total of 61 participants (age: mean 71.2, SD 7.03 years) self-assessed their own homes using the digital self-assessment tool. The overall levels of agreement were high, supporting the validity of the tool in identifying potential hazards. Lower levels of agreement were found in the following domains: steps (77% agreement, Gwet AC_1_=0.56), toilets (56% agreement, κ=0.10), bathrooms (64% agreement, κ=0.46), and backyards (55% agreement, κ=0.24).

**Conclusions:**

Older people were able to self-assess their homes using a digital self-assessment tool. Digital health tools enable older people to start thinking about their future housing needs. Innovative tools that can identify problems and generate solutions may improve the age-friendliness of the home environment.

## Introduction

### Background

Worldwide, people are living longer because of increased life expectancy and declining fertility rates [1]. Recent data show that the number of older people aged >60 years will increase from 1 billion in 2020 to 1.4 billion in 2030 [2]. By 2050, the number of older people aged ≥60 years will double, reaching 2.1 billion [2]. Aging leads to changes in intrinsic capacity (eg, physical and cognitive abilities) and functional ability. In turn, the environment in which the person lives may require adaptation. Over time, the home environment must be able to support a decline in both intrinsic capacity and functional ability [3].

The establishment of age-friendly environments in homes and communities will play an important role in optimizing the health and well-being of society [4]. Most people want to remain in their own homes as they age [5,6]. A survey of >10,000 Australians found that 80% of older people wanted to remain in their current homes [7]. Approximately 20% of older people preferred to live in long-term facilities [7]. In Canada, a population-wide survey of people aged 65 years found that >70% had not moved in the past 5 years [8,9]. In Hong Kong, research has found a strong preference among older people to stay at their homes, with family members, or in places that are familiar with their living environment [10]. Aging in place refers to the ability to remain at home for as long as possible, despite a decline in functional ability [11]. Supporting adults to age in place requires consideration of the house as not only a building but also a home [11]. Older people feel strongly connected with their homes and communities, as they provide security, comfort, and a place for self-reflection [5]. Homes are also considered a place to cherish memories and maintain a sense of belonging, which prevents loneliness [12,13]. The ability to age in place depends on the appropriateness of the home, the potential to make alterations to the home, cost and availability of suitable housing alternatives, and formal supports [14].

Occupational therapists often conduct home environment assessments and recommend modifications to improve safety and function in older people and reduce the risk of falls [15]. Examples of home modifications are the installation of grab rails in the shower, decluttering of overcrowded bedrooms, and installation of threshold ramps to eliminate trip hazards within the home [16]. Despite the proven benefits of home environment assessments, access is limited, particularly in rural areas, and assessments are usually available only after injury or illness [17-19]. Home environment assessments can take considerable time, averaging 80 minutes per home assessment [20]. Older adults have identified the potential benefits of adaptations and modifications earlier in the course of aging [21] and are receptive to more education about actions that can be taken to support aging in place [22].

To date, most home environment assessment tools have been developed for administration by occupational therapists [23]. Furthermore, most tools have been developed for use with older people with impaired functional ability, rather than those who are considering future needs to support aging in place [23]. A recent review of home accessibility assessment tools identified 7 home accessibility assessment tools that were considered promising; however, none of the tools had strong evidence supporting reliability and validity [24]. In recent years, home self-assessment tools have emerged. Ziebart et al [25] described the development of a self-assessment checklist that could be used by older adults to assess fall risks in the house [25]. The Home Safety Self-Assessment is another tool that includes a self-assessment checklist and has been shown to have good reliability and validity [26]. Further research aimed at developing and validating tools that can be used by older people to assess their homes to prepare for aging in place is required.

### This Study

This project is part of a research program that seeks to develop a digital health tool to enable middle-aged and older people to self-assess their own homes to understand how to improve the accessibility and age-friendliness of the home environment. The tool was co-designed with older people and developed into a prototype. This study aimed to determine the validity of a home environment self-assessment tool and investigate the levels of agreement between completion by an occupational therapist and completion by an older adult. The research questions were as follows: (1) is it feasible for older people to self-assess their own home environment using a digital health tool? and (2) what are the levels of agreement between an occupational therapist and older person when using a home environment assessment tool?

## Methods

### Study Design

This study involved recruiting a cohort of older adults who completed the home environment self-assessment tool at the same time as an occupational therapist. The study design was used to establish the agreement (validity) of the self-assessment tool by assessing it against the current gold standard, an occupational therapy home assessment. This study was conducted across metropolitan Adelaide, South Australia, Australia.

### Ethics Approval

This study was approved by the Flinders University Human Research Ethics Committee (project number 5303).

### Participants

Participants were recruited if they met the following criteria: (1) being aged ≥60 years, (2) living within their own home either in a private dwelling or in a retirement village, (3) not having a significant level of disability (measured using the Modified Rankin Score, where people must score 2, which indicates that the participant is “able to carry out all usual duties and activities” or “unable to carry out all previous activities but able to look after own affairs without assistance”) [27]. Participants were included if they were aged ≥60 years. Although the ages of 60 to 65 years are not classified as older age, it is at these ages that many people plan retirement and consider longer-term living options [28].

### Recruitment

The participants were recruited from June to November 2022 through local council newsletters, the research department’s registry of interested participants, and existing research networks. Individuals who expressed interest were contacted by the lead researcher (RD) via phone or email. They were provided with a copy of the participation information sheet and a written consent form. The included participants were offered an honorarium in recognition of their time (Aus $20; US $13) and a copy of their self-assessment results and the occupational therapy home assessment results at the end of the study.

### Instrument

The self-assessment tool was specifically developed for this research program based on a review of existing tools and co-design workshops with older people [29]. In this study, the tool was made available via a website and displayed on a tablet computer (iPad; Apple, Inc). The self-assessment tool was developed using a co-design process led by an occupational therapist (KL) and built by a website designer. The tool contains 89 questions within the following domains: general safety, cleaning and maintenance, front entry and garden, hallways, kitchen, toilets, bedrooms, living rooms, bathrooms, laundry, backyard, internal steps, and neighborhood. Each question comprised the following possible responses: yes (satisfactory), no (needs improvement), and not applicable (not present). Participants also answered demographic questions related to their socioeconomic status; marital status; level of education; living status; housing type; ownership of housing; community services received; and whether they considered relocating in the future, which was recorded as a categorical variable, that is, as yes, no, or not applicable. In this study, a maximum of 2 of each area were assessed and data presented for ease of reporting (eg, 2 bathrooms and 2 bedrooms).

### Data Collection

Potentially eligible participants were screened against the eligibility criteria by the lead researcher and occupational therapist, as mentioned earlier. Upon obtaining consent via phone or email, a time and date were scheduled for each participant to complete the self-assessment and receive the standardized occupational therapy home assessment. Before each visit, an offsite previsit risk assessment was completed to ensure that there were no specific safety risks to the therapist and research assistant (eg, COVID-19 infection).

### Self-Assessment Procedure

At each visit, the occupational therapist demonstrated the use of the self-assessment tool using a study iPad with an inbuilt Wi-Fi card for internet access. The occupational therapist used an adapted version of the self-assessment program using a second iPad (which reflects that the therapist is the administrator, rather than the participant). A copy of the self-assessment tool is available on the internet [30]. The participant and occupational therapist simultaneously completed the self-assessment using the study iPads. The participant and the occupational therapist walked through the home together, did not discuss the content of the assessment, and scored each question independently. If 2 people were living in the same house, the occupational therapist and research assistant ensured that the 2 people were not sharing answers to limit bias.

### Data Analysis

Data were entered into Microsoft Excel (Microsoft Corp) and exported to SPSS (IBM Corp) and Stata (StataCorp) software [31,32]. Descriptive statistics were used to report categorical and continuous variables, including the participants’ demographic characteristics, responses to questions regarding the type of housing they lived in, the ownership of their home, formal services received, their postcode, whether they considered relocating in the future, and level of confidence using digital technology. Socioeconomic status was categorized according to the Australian Bureau of Statistics Index of Relative Socioeconomic Advantage and Disadvantage [33]. Each socioeconomic area was given a score (eg, Statistical Area Level 1). The scores ranged from a low index score (more disadvantaged, Statistical Area Level 1) to a high index score (most advantaged, Statistical Area Level 5) [33].

The rooms of each home were assessed using a series of questions related to home safety, which could be given a “yes/no” response. A total of 7 possible responses were developed: yes-yes, no-no, no-yes, yes-no, not applicable-not applicable, yes-not applicable, and no-not applicable. The κ statistic measure of agreement was used to examine the interrater reliability and level of agreement between the participant and occupational therapist using the same self-assessment tool. The level of agreement was determined through individual items. κ scores were presented to provide the agreement between the raters. κ scores ranged from 0, which represented no agreement beyond what can be expected by chance, to 1, which represented perfect agreement between the raters [34]. For this analysis, Cohen κ guidelines of interpretation were applied as suggested by McHugh [34]; values 0 to 0.20 indicated no agreement, 0.21 to 0.39 indicated minimal agreement, 0.40 to 0.59 indicated weak agreement, 0.60 to 0.79 indicated moderate agreement, 0.80 to 0.90 indicated strong agreement, and >0.90 indicated almost perfect agreement. This analysis interpreted any κ <0.60, suggesting inadequate agreement between the 2 raters [34].

Variations of κ were used to assess validity. Where the results showed a high agreement but the κ value was low, Gwet AC_1_ was applied. Dettori and Norvell [35] suggested that there are limitations to κ; high agreement can result in low κ [35,36], and κ values depend on sample sizes, the number of categories, and distribution of responses. Gwet AC_1_ was used to overcome these problems [35]. Wongpakaran et al [37] recommended that Gwet AC_1_ be considered for interrater reliability analyses alongside Cohen κ.

## Results

### Participants

A total of 61 participants completed the self-assessment tool. Table 1 presents the demographic characteristics of these 61 participants. The mean age of the participants was 71.2 (SD 7.03) years. The sample consisted of slightly more female participants (34/61, 56%) than male participants (27/61, 44%). Among the 61 participants, 39% (n=24) lived with a spouse or family member and conducted the self-assessment independently but within the same home at the same time. A total of 59 (97%) participants were assessed as having no disability, 1 (2%) participant had no significant disability despite symptoms, and 1 (2%) participant had a slight disability but was able to look after their own affairs without assistance [27]. All self-assessments were conducted in metropolitan Adelaide. Houses that were considered as “other” were homes built within retirement villages or were defined as apartments by the participant. Almost all the participants (60/61, 98%) did not use a mobility aid at home.

**Table 1:** Demographic characteristics of the participants (N=61).

Demographics			Values
Age, mean (SD; range)			71.2 (7.03; 60-88)
**Sex, n (%)**			
	Male	27 (44)	
	Female	34 (56)	
**Level of education, n (%)**			
	High school	9 (15)	
	Higher education	44 (72)	
	Other	6 (10)	
**Marital status, n (%)**			
	Married	40 (66)	
	Not married	21 (34)	
**Living status, n (%)**			
	Alone	15 (25)	
	Living with spouse	38 (62)	
	Living with family member	6 (10)	
	Other	1 (2)	
**Type of housing, n (%)**			
	House	50 (82)	
	Town house	1 (2)	
	Unit	4 (7)	
	Other	6 (10)	
**Ownership, n (%)**			
	Private owner	57 (93)	
	Private rental	1 (2)	
	Other	3 (5)	
**Services, n (%)**			
	Cleaning and gardening	2 (3)	
	Cleaning	3 (5)	
	Gardening	2 (3)	
	None	51 (84)	
**Socioeconomic status, n (%)**			
	SA^a^1 (most disadvantaged)	5 (8)	
	SA2	6 (10)	
	SA3	16 (26)	
	SA4	21 (34)	
	SA5 (least disadvantaged)	13 (21)	
**Considering relocation, n (%)**			
	Yes	2 (3)	
	No	49 (80)	
	Considering	10 (16)	
**Mobility aid use at home, n (%)**			
	Yes	1 (2)	
	No	60 (98)	

^a^SA: Statistical Area.

The average time taken to complete the self-assessment tool was 23 (SD 8.12) minutes. Table 2 shows the responses on the use and confidence in the use of the self-assessment tool. A total of 16 (26%) out of the 61 participants had minor technical difficulties with the use of the self-assessment tool on the iPad. These technical difficulties were due to accidentally exiting the self-assessment application and not knowing how to return to the original screen or being unable to scroll up or down the iPad. Despite technical difficulties, more than half (44/61, 72%) of the participants found the self-assessment tool easy to use.

**Table 2:** Responses to the questions on the self-assessment tool.

Responses to questions		Values
Time taken to complete the self-assessment tool (min), mean (SD; range)		23 (8.12; 11-60)
**Was the self-assessment tool easy to use? (1: hard; 10: easy)**		
	10	44 (72)
	9	14 (23)
	8	1 (2)
	7.5	1 (2)
	7	1 (2)
**Were there any technical difficulties?**		
	Yes	16 (26)
	No	45 (74)

### Levels of Agreement

#### Overview

Most participants (52/61, 85%) lived in homes with ≥2 bedrooms, 2 bathrooms, and 1 living room. In homes with >2 bedrooms, one of the bedrooms was used as a study room, for guests, for storage, or for grandchildren. Only 18% (11/61) of the homes had internal steps. Among the 61 participants, 11 (18%) had a 3-bedroom house, 7 (11%) had a 4-bedroom house, and only 2 (3%) had a 5-bedroom house. Most participants (60/61, 98%) had a backyard or shared outdoor space within a retirement village or an apartment.

An overview of the levels of agreement between each participant and occupational therapist is outlined in Tables 3 and 4. Overall, all the “general” and “neighborhood”-related questions demonstrated an almost perfect agreement, as all these questions became a point of discussion related to the opinion of the participant.

**Table 3:** Agreement levels for cleaning, front access, hallways, and kitchen between the occupational therapist and participant.

Domain and questions asked between raters			Agreement (%)		Cohen κ		Gwet AC_1_		95% CI
**Cleaning**									
	1. Are clotheslines easy to access (height and location)?	85		0.34^a^		0.81		0.76-0.94	
	2. Is there an irrigation system in place with ease of watering?	93		0.89		0.91		0.87-1.00	
	3. Are there strategies to change lightbulbs, access high cupboards and clean gutters which don’t require use of a ladder?	89		0.69^a^		0.86		0.80-0.97	
	4. Are long lasting lightbulbs (LED) installed to reduce the need for frequent changing?	97		0.66^a^		0.97		0.92-1.00	
	5. Is the home largely clutter free?	90		0.75		0.84		0.82-0.98	
	6. Is there a supportive step stool available to access items which are just out of reach	95		0.70		0.95		0.90-1.00	
**Front access**									
	1. Are paths relatively flat and approximately 1000mm wide?	72		0.19^a^		0.60		0.61-0.84	
	2. Do paths and driveways have a non-slip texture and are they free of moss?	79		0.24^a^		0.71		0.68-0.89	
	3. Is the gate easy to open?	85		0.68^a^		0.81		0.76-0.94	
	4. Are steps a suitable height (115-190mm) and depth (240mm-355mm) and stable?	79		0.51		0.73		0.68-0.89	
	5. Is it easy to unlock the front door and use the door handle?	89		0.33^a^		0.86		0.80-0.97	
	6. Is a lockable screen door in place to enable access to fresh air and maintain security?	87		0.57^a^		0.85		0.78-0.96	
	7. Is there space within the garage or carport to easily open the car door and get out?	93		0.57^a^		0.93		0.87-1.00	
	8. Is the letterbox easy to access and open?	75		0.00^a^		0.69		0.64-0.87	
	9. Is there at least one way to access the home without a step?	77		0.56		0.54		0.66-0.88	
**Hallways**									
	1. Are hallways free of clutter and unnecessary furniture?	87		0.50^a^		0.82		0.78-0.96	
	2. Are floor coverings secure and in good condition?	79		0.36^a^		0.74		0.68-0.89	
	3. Is the house free of internal steps?	97		0.91		0.95		0.92-1.00	
**Kitchen**									
	1. Is there room within the kitchen to easily manoeuvre?	100		1.00		1.00		1.00-1.00	
	2. Are benches clear?	82		0.38^a^		0.75		0.72-0.92	
	3. Are rugs and floor coverings secure and in good condition?	79		0.46		0.74		0.68-0.89	
	4. Are you able to easily reach or commonly used items without tiptoes, a stepladder, or bending too low?	92		0.40^a^		0.91		0.85-0.99	
	5. Are taps easy to turn on, off and adjust?	98		0.00^a^		0.98		0.95-1.00	
	6. Can appliance controls easily be accessed?	100		1.00		1.00		1.00-1.00	
	7. Is there space next to the microwave, oven, and stove top to place hot food?	80		0.46^a^		0.70		0.70-0.91	
	8. Is there a carbon monoxide detector installed to detect carbon monoxide and prevent poisoning?	93		0.48^a^		0.93		0.87-1.00	
	9. Is there a space in the kitchen areas where you could sit if needed to prepare food?	89		0.18^a^		0.87		0.80-0.97	
	10. Are stools a comfortable height and stable?	84		0.70		0.78		0.74-0.93	
	11. Are the oven and microwave located at a suitable height? With Access between knee and shoulder?	77		0.23^a^		0.68		0.66-0.88	
	12. Are bench tops a suitable height (850mm to 1050mm)?	100		1.00		1.00		1.00-1.00	

^a^Where the results showed a high agreement but the κ value was low, Gwet AC_1_ was applied.

**Table 4:** Agreement levels for internal steps, bathroom, toilet, bedroom, lounge area, laundry, and backyard between the occupational therapist and participant.

Domain and questions asked between raters			Agreement (%)		Cohen κ		Gwet AC_1_		95% CI
**Internal step**									
	1. Do internal stairs have a sturdy rail in place?	91		0.62		0.88		0.71-1.00	
	2. Are doorways a minimum of 850mm wide?	100		1.00		1.00		1.00-1.00	
	3. Are door handles lever style?	82		0.68		0.75		0.55-1.00	
	4. Can doors and windows be easily opened to allow for fresh air?	73		−0.06^a^		0.68		0.41-1.00	
**Bathroom 1**									
	1. Is there room within the bedroom to easily manoeuvre?	78		0.29^a^		0.67		0.66-0.88	
	2. Are rugs or mats secure and in good condition?	64		0.46		0.48		0.52-0.76	
	3. Is there adequate ventilation with presence of a fan or easily opened window?	100		1.00		1.00		1.00-1.00	
	4. Is the transition between the floor and shower flat?	61		0.27		0.22		0.48-0.73	
	5. Is a shower hose in place?	82		0.84		0.89		0.85-0.99	
	6. Are taps easy to turn on, off and adjust?	66		0.08^a^		0.49		0.53-0.78	
	7. Is water thermostatically controlled to a delivery temperature of 45 degrees?	84		0.70		0.77		0.74-0.93	
	8. Is the floor surface non-slip?	67		0.33		0.38		0.55-0.79	
	9. Is the shower cubicle a minimum of 900×900mm?	92		0.70		0.90		0.85-0.99	
**Bathroom 2**									
	1. Is there room within the bathroom to easily manoeuvre?	56		0.14		0.25		0.42-0.71	
	2. Are rugs or mats secure and in good condition?	46		0.29		0.19		0.31-0.61	
	3. Is there adequate ventilation with presence of a fan or easily opened window?	94		−0.04^a^		0.93		0.86-1.00	
	4. Is the transition between the floor and shower flat?	54		0.20		0.11		0.39-0.69	
	5. Is a shower hose in place?	92		0.84		0.89		0.83-1.00	
	6. Are taps easy to turn on, off and adjust?	63		0.14^a^		0.40		0.48-0.77	
	7. Is water thermostatically controlled to a delivery temperature of 45 degrees?	90		0.80		0.86		0.80-1.00	
	8. Is the floor surface non-slip?	71		0.33		0.49		0.57-0.84	
	9. Is the shower cubicle a minimum of 900×900mm?	79		0.41		0.75		0.67-0.92	
**Toilet 1**									
	1. Is the toilet a suitable height (460mm-480mm)?	55		0.10		0.27		0.40-0.71	
	2. Are rugs or mats secure and in good condition and necessary?	70		0.53		0.58		0.59-0.82	
	3. Does the door swing outwards?	90		0.79		0.87		0.82-0.98	
**Toilet 2**									
	1. Is the toilet a suitable height (460mm-480mm)?	55		0.01^a^		0.27		0.40-0.71	
	2. Are rugs or mats secure and in good condition and necessary?	62		0.42		0.45		0.46-0.80	
	3. Does the door swing outwards?	89		0.79		0.86		0.78-1.00	
**Bedroom 1**									
	1. Is the bed a comfortable height to access and rise from?	93		0.31^a^		0.93		0.87-1.00	
	2. Is there space to easily manoeuvre within the bathroom?	75		0.16^a^		0.67		0.64-0.87	
	3. Is there access to light and phone next to the bed?	97		0.65^a^		0.96		0.92-1.00	
	4. Are floor covering secure and in good condition?	84		0.38^a^		0.81		0.74-0.93	
	5. Is there somewhere to sit while dressing and putting on shoes?	92		0.51^a^		0.91		0.85-0.99	
	6. Is it easy to access clothing and shoes without excessive reaching or bending?	93		−0.03^a^		0.93		0.87-1.00	
	7. Is it easy to open and close windows and blinds?	85		0.12^a^		0.82		0.76-0.94	
	8. Can the temperature in the bedroom be easily adjusted?	92		0.72		0.90		0.85-0.99	
**Bedroom 2**									
	1. Is the bed a comfortable height to access and rise from?	85		0.39^a^		0.83		0.74-0.95	
	2. Is there space to easily manoeuvre within the bedroom?	81		0.47^a^		0.77		0.70-0.92	
	3. Is there access to light and phone next to the bed?	90		0.41^a^		0.90		0.82-0.99	
	4. Are floor covering secure and in good condition?	90		0.58^a^		0.89		0.82-0.99	
	5. Is there somewhere to sit while dressing and putting on shoes?	94		0.65^a^		0.93		0.88-1.00	
	6. Is it easy to access clothing and shoes without excessive reaching or bending?	96		0.73^a^		0.96		0.91-1.00	
	7. Is it easy to open and close windows and blinds?	75		0.22^a^		0.64		0.63-0.87	
	8. Can the temperature in the bedroom be easily adjusted?	90		0.62		0.89		0.82-0.99	
**Living area 1**									
	1. Is there space to easily manoeuvre within the living area?	87		0.29^a^		0.84		0.78-0.96	
	2. Are floor covering secure and in good condition?	80		0.09^a^		0.78		0.70-0.90	
	3. Is there good storage so that all items have a spot?	95		0.38^a^		0.95		0.90-1.00	
	4. Is the room free of cords in walkways which may cause trips?	97		0.00^a^		0.97		0.92-1.00	
	5. Is it easy to access heating and cooling controls?	93		0.53^a^		0.93		0.87-1.00	
	6. Is it easy to open and close windows and blinds?	90		−0.03^a^		0.89		0.82-0.98	
	7. Are chairs in the room easy to get in and out of?	44		0.02		0.04		0.31-0.57	
**Living area 2**									
	1. Is there space to easily manoeuvre within the living area?	87		0.65		0.78		0.73-1.00	
	2. Are floor covering secure and in good condition?	73		0.13^a^		0.69		0.57-0.90	
	3. Is there good storage so that all items have a spot?	87		0.52^a^		0.84		0.74-1.00	
	4. Is the room free of cords in walkways which may cause trips?	90		0.37^a^		0.88		0.79-1.00	
	5. Is it easy to access heating and cooling controls?	100		1.00		1.00		1.00-1.00	
	6. Is it easy to open and close windows and blinds?	77		0.15^a^		0.73		0.61-0.93	
	7. Are chairs in the room easy to get in and out of?	53		0.07^a^		0.42		0.34-0.73	
**Laundry**									
	1. Is there adequate bench space in the laundry?	84		0.65		0.79		0.74-0.93	
	2. Can all appliances be easily accessed and plugged in when needed?	85		0.40^a^		0.83		0.76-0.94	
	3. Is there room in the house to hang small items of laundry to dry when needed?	97		0.78		0.96		0.92-1.00	
	4. Is the washing machine front-loading?	100		1.00		1.00		1.00-1.00	
**Back garden**									
	1. Are paths relatively flat and approximately 1000mm wide?	78		0.24^a^		0.75		0.68-0.89	
	2. Are doorways a minimum of 850mm wide?	100		1.00		1.00		1.00-1.00	
	3. Is it possible to access the clothesline without excessive reaching?	63		0.29^a^		0.52		0.51-0.76	
	4. Is the garden low maintenance in terms of watering requirements, lawn mowing and management of autumn leaves?	55		0.24		0.39		0.42-0.68	
	5. Are there shady areas outside to sit?	93		0.57^a^		0.92		0.87-1.00	
	6. Is outdoor furniture sturdy, comfortable and easy to get on/off?	83		0.40^a^		0.81		0.74-0.93	

^a^Where the results showed a high agreement but the κ value was low, Gwet AC_1_ was applied.

Among the 61 participants, the domains that demonstrated the lowest agreement levels between the occupational therapist and participant were the front garden and entry (72% to 93% agreement), bathrooms (46% to 100% agreement), toilets (54% to 92% agreement), and backyards (55% to 100% agreement).

#### Front Access

Items that showed a moderate level of agreement were paths being flat and wide (72% agreement, Gwet AC_1_=0.60), paths and driveways having a nonslip texture and being free of moss (79% agreement, Gwet AC_1_=0.71), and the letter box being easy to access and open (75% agreement, Gwet AC_1_=0.69). Items with weak agreement were related to the front steps of the house. For example, steps being of a suitable height and depth and stable demonstrated 79% agreement (κ=0.51), and whether the home had at least 1 way to access it without a step demonstrated 77% agreement (Gwet AC_1_=0.56). No participant assessed the front steps as being unsuitable and unstable, as opposed to the occupational therapist, who assessed 17 front steps as being unsuitable and unstable.

#### Hallways

The item that showed a strong agreement was the hallways being free of clutter and unnecessary furniture (87% agreement, Gwet AC_1_=0.82). A total of 13 floor coverings within the hallways were assessed by the occupational therapist as being unsafe; by contrast, no participant assessed the floor coverings as being unsafe.

#### Kitchen

The responses to a total of 12 questions regarding the kitchen were compared for levels of agreement between the occupational therapist and participant. There was an overall weak to almost perfect level of agreement, as shown in Table 3. The occupational therapist assessed the oven and microwave to be at an unsuitable height on 14 (23%) out of 61 occasions; by contrast, no participant assessed the oven or microwave to be at an unsuitable height.

#### Bathroom 1

For the 9 questions regarding bathrooms, the levels of agreement varied from minimal to almost perfect, as shown in Table 4.

Both the occupational therapist and participant agreed (100% agreement, κ=1.00) that there was adequate ventilation with the presence of a fan or window. There was moderate agreement (78% agreement, Gwet AC_1_=0.67) for bathroom 1 being easy to maneuver in and for the shower cubicle being a minimum of 900×900 mm in size (92% agreement, κ=0.70). There was a weaker level of agreement for rugs or mats being secure (64% agreement, κ=0.46) and for taps being easy to turn on, turn off, and adjust (66% agreement, κ=0.49). Moreover, there was minimal agreement for the transition between the floor and shower being flat (61% agreement, κ=.27) and for the floor surfaces being nonslip (67% agreement, κ=0.33). Most participants did not believe that “shower lips” and “shower alcove tracks” were home hazards and commonly considered these transitions to be flat.

#### Toilet 1

For the 3 questions regarding toilets, the levels of agreement varied from no agreement to strong agreement, as shown in Table 4. Toilet 1’s height had the lowest agreement (no agreement between the occupational therapist and participant; 56% agreement, κ=0.10) among the items. Both the occupational therapist and participant agreed that the toilet heights were suitable on 31 (51%) out of 61 occasions, whereas on 27 (44%) out of 61 occasions, the occupational therapist assessed the toilet height as being unsuitable. Participants commonly indicated the toilet height as currently manageable and not an area of concern.

#### Bedroom 1

For the 8 questions regarding bedrooms, the levels of agreement varied from moderate to almost perfect, as shown in Table 4.

The participants and occupational therapist both highly agreed that there was easy access to light and phone next to the bed (97% agreement, Gwet AC_1_=0.96). Other items that indicated a high level of agreement were the bed being of a comfortable height to access and rise from (93% [almost perfect] agreement, Gwet AC_1_=0.93), clothing and shoes being easy to access (93% [almost perfect] agreement, Gwet AC_1_=0.93), having a place to sit when dressing and putting on shoes (92% [almost perfect] agreement, Gwet AC_1_=0.91), and the temperature in the bedroom being easily adjustable (92% [almost perfect] agreement, κ=0.72). Although the participants were asked to assess bed heights, most participants (56/92, 61%) interpreted the bed height question as “was the bed comfortable,” rather than whether the bed was at a “comfortable height.”

#### Living Areas

Living areas 1 and 2 were classified by the participants as their main living areas where they watch television, rumpus rooms, or sitting areas.

For the 7 questions regarding living areas, the levels of agreement varied from no agreement to almost perfect agreement, as shown in Table 3.

An almost perfect level of agreement was evident for the following items: the walkways in the lounge area being free of cords (97% agreement, Gwet AC_1_=0.97), the lounge area having good storage capacity (95% agreement, Gwet AC_1_=0.95), and having easy access to heating and cooling controls (93% agreement, Gwet AC_1_=0.93). A strong agreement level was illustrated for there being enough circulation space (90% agreement, Gwet AC_1_=0.84) and for the windows or blinds being easy to open (90% agreement, Gwet AC_1_=0.89) within the living area.

Floor coverings in the lounge area seemed to indicate “lower” levels of agreement (80% [moderate] agreement, Gwet AC_1_=0.78). The occupational therapist disagreed with the participant and indicated that 8 (13%) out of 61 lounges had unsafe floor coverings.

Among the items assessing living area 1, the lowest level of agreement was for whether the chairs in the room were easy to get in and out of (no agreement between the occupational therapist and participant; 44% agreement, κ=0.02).

#### Backyard

For the 6 questions regarding the backyard, the levels of agreement varied from minimal agreement to almost perfect agreement, as shown in Table 3.

There was an almost perfect level of agreement between the participants and occupational therapist for whether the back garden doorway was a minimum of 850 mm wide (100% agreement, κ=1.00) and for whether there were shady areas outside to sit (93% agreement, Gwet AC_1_=0.92). Whether the outdoor furniture was sturdy, comfortable, and easy to get on and off also had a high level of agreement (83% [strong] agreement, Gwet AC_1_=0.81).

There was a moderate level of agreement for the paths being relatively flat and approximately 1000 m wide (78% agreement, Gwet AC_1_=0.75). Here, the occupational therapist disagreed on 9 (15%) out of 60 occasions, and there was agreement between the raters on only 2 (3%) occasions. Among the items regarding the backyard, the lowest agreement levels were observed for whether it was possible to access the clothesline without excessive reaching (63% [weak] agreement, Gwet AC_1_=0.52) and whether the garden was low maintenance (κ=0.24, minimal agreement with 55%).

## Discussion

### Principal Findings

In this study, a digital home environment self-assessment tool was tested with older people, and its validity was determined through an assessment of the levels of agreement between an occupational therapist and older person. The overall levels of agreement were high, supporting the validity of the tool in identifying potential hazards. Lower levels of agreement were found between the occupational therapist and older participants in the following domains: steps, toilets, bathrooms, and backyards. Items regarding the height of toilets; height of chairs in the lounge; loose rugs, mats, or floor coverings; height of kitchen appliances; and transition between shower alcoves and bathroom flooring also displayed lower levels of agreement. Lower levels of agreement likely occurred owing to (1) the subjective nature of some questions, such as “is there at least one way to access the home without a step?” and (2) the more critical lens through which an occupational therapist assesses the home environment. There were no items where participants were more likely to identify hazards than the occupational therapist.

Participants found the tool to be relatively simple and quick to complete, and overall, there were high levels of agreement. The study conducted by Ali and Kumar [38] also found that older people were able to self-assess potential risk factors at home. They also found that self-assessments led to older people being able to initiate minor modifications to their homes, including the removal of throw rugs and the reorganization of kitchen appliances. Other research has shown that older people prefer self-assessment approaches that go beyond identifying hazards and provide them with potential solutions to ensure that their home is safe and comfortable [39]. Checklists and recommendations for improving the age-friendliness of the home environment may also be useful for architects and designers to help them gain insights into the practical needs of older people.

Although older people were able to self-assess their homes, there were often occasions of disagreement between the perspective of the occupational therapist and that of the participant. In particular, the assessments of steps, toilets, bathrooms, and the backyard showed conflicting results. Occupational therapists have extensive training in environmental assessments with an emphasis on safety [29]. Another study has also shown that occupational therapists are more critical of the environment than other people [40]. Lower levels of agreement were commonly observed for items assessing bathrooms and toilets. These areas have been shown to be particularly hazardous for older people. Gell et al [41] found that bathroom modifications were common and usually increased after multiple falls. Similarly, Wellecke et al [42] found that bathroom modifications were frequently required to support aging in place. Their study also found that large step-free showers and bathrooms on the ground floor were beneficial [42]. It was clear from the participants in this study that bathrooms and toilets were not an area of concern for them yet. Bathroom and toilet modifications, such as the addition of grab rails, may be a key feature to consider in the design of new buildings in an age-ready city.

We also found a difference in agreement levels for items regarding the backyard, with many participants indicating that their gardens did not require high maintenance, despite the occupational therapist believing they did. Research shows that gardening stimulates a greater level of well-being, better physical and mental health, and better sleep quality among older people [43]. However, as aging takes place, older people have also described concerns about maintaining large gardens [44]. Suitable gardening solutions, such as landscaping options and irrigation systems for reducing maintenance, may facilitate age-friendly environments. Given that most older people experience a sense of connection with their homes [12,13], practical support for gardening or access to parks and gardens within walking distance can support the development of healthy aging cities.

### Limitations

This study has several limitations that should be acknowledged. Participants were recruited through a variety of methods, and the use of convenience sampling may have influenced the results, as the population was not representative of the general population. Most participants (50/82, 61%) lived in metropolitan areas with high socioeconomic status. These living conditions may differ from those in other countries and those of other older populations. Further research should include participants with a lower socioeconomic status and those living in rural or remote areas.

The results may have been influenced by the variations in the interpretation of the questions. For example, some participants indicated that some questions were ambiguous. For example, the interpretation of the question “is the home largely clutter free?” depended on the person’s perception of clutter. Some of our participants (eg, spouses or family relatives) lived in the same house; however, the assessments were completed independently and without consultation between the cohabitants. Finally, our CIs may have been narrower with a larger sample size.

### Conclusions

In conclusion, older people were able to self-assess their own homes using a digital health tool. The purpose of the digital tool was to enable people to start thinking about future housing decisions. This study showed that although agreement levels were generally high, older people and occupational therapists may still have different views on the safety of home environments. In particular, the items regarding steps, toilets, bathrooms, and backyards were subject to different perspectives. Following this research, the digital tool will be slightly modified to address questions for which there was a higher level of disagreement. Attempts will be made to reduce the ambiguity of some questions. Tools that identify potential problems and generate solutions are likely to be of value in supporting future housing decisions as populations age.
